# Healthcare use for acute gastrointestinal illness in two Inuit communities: Rigolet and Iqaluit, Canada†

**DOI:** 10.3402/ijch.v74.26290

**Published:** 2015-05-21

**Authors:** Sherilee L. Harper, Victoria L. Edge, James Ford, M. Kate Thomas, David Pearl, Jamal Shirley, IHACC, RICG, Scott A. McEwen

**Affiliations:** 1 Department of Population Medicine, University of Guelph, Guelph, Ontario, Canada; 2 Office of Public Health Practice, Public Health Agency of Canada, Guelph, Ontario, Canada; 3 Department of Geography, McGill University, Montreal, Quebec, Canada; 4 Centre for Food-borne, Environmental & Zoonotic Infectious Diseases, Public Health Agency of Canada, Guelph, Ontario, Canada; 5 Nunavut Research Institute, Iqaluit, Nunavut, Canada; 6 Indigenous Health Adaptation to Climate Change Research Group: Lea Berrang-Ford, Cesar Carcamo, Alejandro Llanos, Shuaib Lwasa, Didacus Bambaiha Namanya; 7 Rigolet Inuit Community Government, Rigolet, Nunatsiavut, Labrador, Canada

**Keywords:** Aboriginal health, Indigenous health, Inuit health, gastrointestinal illness, healthcare utilization, Nunatsiavut, Nunavut

## Abstract

**Background:**

The incidence of self-reported acute gastrointestinal illness (AGI) in Rigolet, Nunatsiavut, and Iqaluit, Nunavut, is higher than reported elsewhere in Canada; as such, understanding AGI-related healthcare use is important for healthcare provision, public health practice and surveillance of AGI.

**Objectives:**

This study described symptoms, severity and duration of self-reported AGI in the general population and examined the incidence and factors associated with healthcare utilization for AGI in these 2 Inuit communities.

**Design:**

Cross-sectional survey data were analysed using multivariable exact logistic regression to examine factors associated with individuals’ self-reported healthcare and over-the-counter (OTC) medication utilization related to AGI symptoms.

**Results:**

In Rigolet, few AGI cases used healthcare services [4.8% (95% CI=1.5–14.4%)]; in Iqaluit, some cases used healthcare services [16.9% (95% CI=11.2–24.7%)]. Missing traditional activities due to AGI (OR=3.8; 95% CI=1.18–12.4) and taking OTC medication for AGI symptoms (OR=3.8; 95% CI=1.2–15.1) were associated with increased odds of using healthcare services in Iqaluit. In both communities, AGI severity and secondary symptoms (extreme tiredness, headache, muscle pains, chills) were significantly associated with increased odds of taking OTC medication.

**Conclusions:**

While rates of self-reported AGI were higher in Inuit communities compared to non-Inuit communities in Canada, there were lower rates of AGI-related healthcare use in Inuit communities compared to other regions in Canada. As such, the rates of healthcare use for a given disease can differ between Inuit and non-Inuit communities, and caution should be exercised in making comparisons between Inuit and non-Inuit health outcomes based solely on clinic records and healthcare use.

Globally, there are substantial disparities in health outcomes between Indigenous and non-Indigenous peoples; these disparities span most indicators of health and wellbeing (1). Contributing to poor health outcomes is the often lower healthcare use and access among a number of Indigenous populations globally (2). A Canadian study comparing Indigenous to non-Indigenous populations found many indicators of use, access and quality of healthcare services to be significantly worse for Indigenous populations, including significantly lower numbers visiting a physician, perceived lower healthcare service accessibility and significantly higher reported unmet healthcare needs for Indigenous people (3). To address these inequities in healthcare, it is important to better understand healthcare use and health practices specific to Indigenous populations to help inform and further develop appropriate primary and public healthcare that will enhance accessibility and quality of care and reflect demand.

In the case of acute gastrointestinal illness (AGI), healthcare use research has informed public health surveillance and medical services at national and provincial levels (4–7). AGI includes diarrhoea and/or vomiting caused by a variety of conditions that cause acute gastrointestinal symptoms, including infections with pathogens transmitted by person-to-person contact or contaminated food or water. For a case of AGI to be captured by a national surveillance system, the case has to come in contact with the healthcare system. For instance, in Canada, the case must visit a healthcare provider, the healthcare provider must request a stool sample, the case must comply and submit a stool sample for testing, the sample must be tested, the sample must test positive for a pathogen that is deemed reportable, and positive cases must be reported to provincial and national health authorities (8). Any break in this chain of events will result in the case not being captured by surveillance efforts; as such, cases of AGI are under-ascertained by surveillance systems due to under-diagnosis and under-reporting (4, 5, 8–10). The under-ascertainment of infectious AGI cases in surveillance systems has important implications for program planning, resource prioritization, and outbreak detection and management (4, 5, 8–10). Therefore, several studies have been conducted within communities to estimate the incidence of AGI in the general population compared to the incidence rates captured by surveillance systems, which allows health authorities to account for under-ascertainment and adjust interpretations of AGI surveillance data (4, 5, 8–10).

The decision to treat AGI symptoms at home and/or seek healthcare depends on a number of factors, including severity of illness, primary and secondary symptoms experienced, demographic characteristics, engagement with and perceptions of healthcare and illness, among others (5, 9). It is important to understand what factors are associated with AGI healthcare use, as well as the similarities and differences between the types of cases captured by health systems compared to those cases who are not captured by health systems. This improves our understanding of what makes someone more or less likely to be counted in reportable disease statistics (4, 5, 8–10); in other words, who is counted and why. This information is valuable to inform and improve medical service provision and adjust for biases in surveillance data that are used for public health planning, programming, monitoring and practice. This research is typically conducted at the national or city level, and generally has not extended to subpopulations experiencing disparities in health outcomes, including Indigenous peoples.

In Canada, when using data from clinic records and surveillance systems there seems to be little difference between laboratory-confirmed AGI rates in northern Indigenous communities compared to non-Indigenous southern communities (11–13); however, when using data from surveys of the general population, self-reported AGI (not necessarily laboratory confirmed) was much higher in 2 northern Indigenous communities compared to similar studies in non-Indigenous communities in Canada and abroad (14) (Fig. 1). This difference suggests that Indigenous communities might interact with the healthcare system differently than other non-Indigenous communities for AGI symptoms. The contradiction between the relatively low incidence of AGI cases identified by surveillance systems and the relatively high incidence of self-reported AGI in Inuit communities has implications for AGI-related primary healthcare and public health practice, as well as decisions that are based on national surveillance data. Therefore, this study examined self-reported medication and healthcare use related to AGI in 2 Inuit communities. Specifically, this study used cross-sectional community surveys to (a) describe symptoms, severity and duration of self-reported AGI, (b) examine and compare the proportion of cases consulting with healthcare professionals with the proportion of stool samples requested and submitted (e.g. the level of AGI under-diagnosis) and (c) identify factors associated with medication and healthcare use for AGI symptoms in 2 Inuit communities: Rigolet, Nunatsiavut, and Iqaluit, Nunavut.

**Fig. 1:**
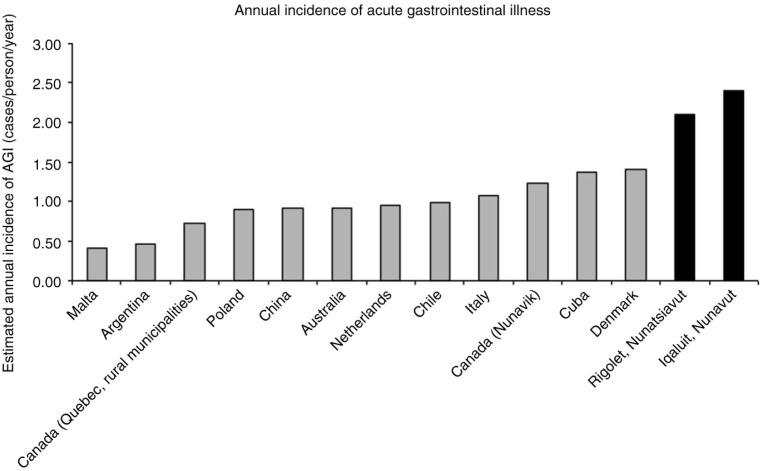
The estimated annual incidence of acute gastrointestinal illness (case definition: 3 or more loose stools/day and/or vomiting in the past 28 days) for Malta (20), Argentina (21), Quebec (22), Poland (23), China (24), Australia (25), Netherlands (7), Chile (26), Italy (6), Nunavik (27), Cuba (28), Denmark (29), Rigolet (14) and Iqaluit (14).

## Methods

### Study locations

**Fig. 2:**
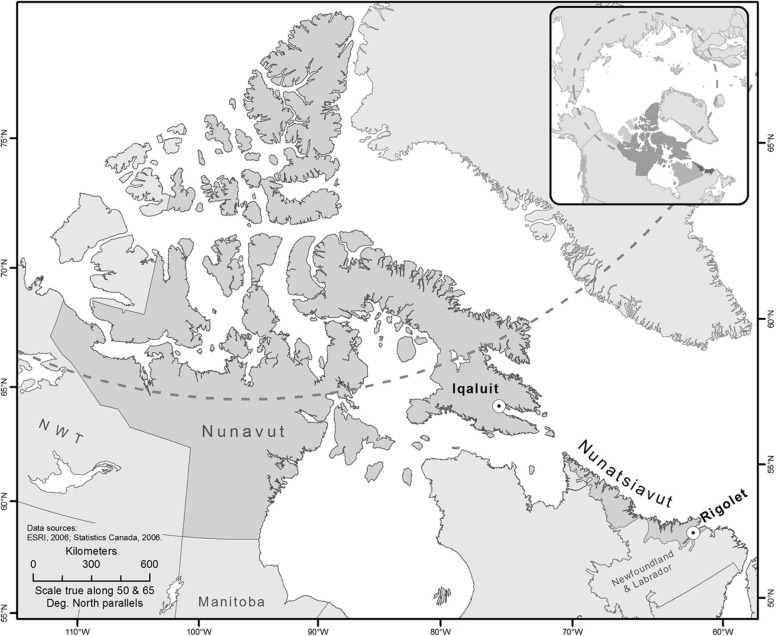
A map displaying the 2 partner study communities: Rigolet, Nunatsiavut, and Iqaluit, Nunavut, Canada.

This study was part of a larger study that found high incidence rates of AGI in Iqaluit, Nunavut, and Rigolet, Nunatsiavut, Labrador, compared to other regions in Canada (14, 15). We partnered with these 2 communities based on prior research relationships and community-identified research interests. By working with 2 communities, we attempted to capture information from a small and large Inuit settlement, urban and remote setting, and from 2 Inuit regions. Iqaluit is the capital city of Nunavut (Fig. 2) and has 6,699 residents, primarily Indigenous people (62%) (16). In Iqaluit, the Qikiqtani General Hospital and the Public Health Centre are staffed with physicians and nurses. Patients requiring more specialized services are flown south for treatment. Healthcare services are paid by the Government of Nunavut's *Nunavut Health Care Plan* for all residents, and the services not covered by the Territorial Plan are paid by the Noninsured Health Benefits (NIHB) Program for Inuit beneficiaries, which is paid by the Federal Government and administered by the Territory. The Extended Health Benefits Program provides coverage for non-Inuit residents. The Government of Nunavut and Federal Government also provide broad health coverage (e.g. Great West Life) to their employees in all Nunavut communities. Rigolet is a small Inuit community located on the north-east coast of Labrador in the province of Newfoundland and Labrador (Fig. 2), with approximately 269 residents, 94% of whom identify as Indigenous (16). The community's health clinic has 2 resident nurses and a visiting physician (every 6 weeks). When necessary, patients are medically evacuated by air to the Labrador Health Center in Goose Bay, Labrador. Medical services are paid for by the Province of Newfoundland and Labrador's *Medical Care Plan*, as well as the NIHB Program for Inuit beneficiaries which is paid by the Federal Government and administered by the Nunatsiavut Government. In both Iqaluit and Rigolet, the NIHB Program pays for a range of prescription *and* over-the-counter (OTC) medications for Inuit residents if a prescription from a licensed practitioner is obtained.

### Data collection

Data were collected as part of a larger burden of AGI study (14). Two cross-sectional surveys were conducted in both Rigolet and Iqaluit: one survey in September 2012 and one survey in May 2013 in an attempt to capture seasonality of AGI outcomes, risk factors and healthcare use. The case definition that was used for AGI included self-reported vomiting and/or diarrhoea in the past 14 days (September and May surveys) and in the past 28 days (May survey only) (4, 10). In an attempt to capture incident cases, if the date of AGI symptom onset was prior to the 14-day/28-day recall period, the case was excluded. Cases were excluded if the participant believed that their recent AGI symptoms were due to pregnancy, medication use, alcohol/drug use, or diagnosed chronic conditions (e.g. colitis, diverticulitis, Crohn's disease, irritable bowel syndrome, current *H. pylori*, or other diagnosed chronic conditions). Cases were defined as mild (less than 3 loose stools and/or a single episode of vomiting in 1 day or less), moderate (3 or more loose stools and/or 2 or more episodes of vomiting, lasting for less than 1 day) and severe (3 or more loose stools and/or 2 or more episodes of vomiting, lasting for more than 1 day) (17). The case definitions, as well as the mild, moderate and severe classifications, were selected to facilitate comparisons with national and international studies examining the self-reported burden of AGI using similar case definitions and methods (4, 10, 17).

The following data were also gathered: primary and secondary symptoms; consulting a nurse or physician; prescription and OTC medication use; traditional Inuit medication use (e.g. local herbs and teas); traditional Inuit healing practices (e.g. use of country foods); impact of AGI on productivity including missed employment, school and traditional activities (e.g. hunting, fishing, trapping, visiting cabins, carving, crafts and so on); socio-economic indicators [e.g. over-crowding, food security status (18)]; and demographic information (14).

In Rigolet, a census sample was attempted; every individual in every household who was in the community during the study period was invited to participate. In Iqaluit, a target sample size of 498 randomly selected participants for each survey was calculated using a 2% allowable error and a 95% confidence level to detect an expected prevalence of 6% based on a population of 6,184 people (14). To randomly select participants, first houses were randomly selected using a City of Iqaluit Housing Atlas, with at least 2 in-person attempts per house at different times of the day (during the day, and then during weekends or evenings) on different days of the week. Following successful contact, an individual from the household was randomly selected using the last birthday method and invited to participate in the survey. All ages were eligible to participate, and for participants under 12, the parent could act as a proxy respondent. A research licence was obtained from the Nunavut Research Institute, and the study protocol was approved by the research ethics boards at the University of Guelph, McGill University and Health Canada, and the Nunatsiavut Government Research Advisory Committee.

### Data analysis

Only data from individuals fitting the AGI case definition were included in the analyses. Participants responding “unsure” or “refused to answer” were excluded from the analysis of that question. In both communities, there was no significant difference in medication or healthcare use outcomes between September and May surveys (p<0.05). Considering the small number of cases and the lack of statistical difference between surveys, data from the September and May surveys were combined for analyses.

Descriptive statistics were used to examine the symptoms, severity and duration of self-reported AGI (objective 1). The level of under-diagnosis was explored by descriptively comparing the proportions of self-reported cases, cases who reported to visit a healthcare professional, and cases who reported to submit a stool sample (objective 2).

To identify factors potentially associated with healthcare use for AGI symptoms (objective 3), in Rigolet, a series of univariable exact logistic regression models were built to examine unconditional associations between potential risk factor variables and OTC medication use. In Iqaluit, 2 multivariable exact logistic models were built with the following outcome variables: (a) visiting a health clinic or hospital and (b) taking OTC medications for AGI. First, a causal diagram was built to explore and identify potential risk factors of interest based on peer-reviewed literature and biological plausibility. Then, a series of univariable exact logistic regression models were built with risk factor variables of interest (significance levels based on the conditional scores test) (19). Those predictor variables with p<0.20 in the univariable models were considered in the multivariable model using an iterative manual forward-step model building approach. Predictors remained in the model if significant (α<0.05) or if they were identified as a confounder (e.g. inclusion resulted in more than a 30% change in the β-coefficient) (19). To avoid collinearity issues, the correlation between predictors variables was assessed using Spearman rank correlation analysis, using a cut-point value of 70%. If the correlation was above 70%, the most biologically plausible variable was used in the model building process (19). All analyses were conducted using Stata IC (version 11.2).

## Results

In Rigolet a census was attempted: of the 245 people in the community during the September 2012 survey period, a total of 226 people agreed to participate in the survey (92% response rate); of the 249 people in the community during the May 2013 survey, a total of 236 people agreed to participate in the survey (95% response rate). In Iqaluit, 532 (September) and 520 (May) randomly selected participants from randomly selected households completed the questionnaire, yielding response rates of 75 and 55%, respectively. There were 62 participants in Rigolet and 125 participants in Iqaluit who reported AGI symptoms that fit the case definition (Tables I and II). Only these AGI cases were considered in the analysis.

**Table I:** Treatment, duration and severity of acute gastrointestinal illness in Rigolet, Nunatsiavut, in September 2012 and May 2013

	Rigolet			
	
	September and May combined			
	
Treatment, duration and severity	All cases % (95% CI) Mean [range]	Mild cases % (95% CI) Mean [range]	Moderate cases % (95% CI) Mean [range]	Severe cases % (95% CI) Mean [range]
Age				
0–19	24.2% (14.9–36.7%)	37.5% (19.8–59.4%)	64.3% (34.0–86.3%)	25.0% (11.0–47.4%)
20–55	56.5% (43.6–68.5%)	50.0% (29.7–70.3%)	35.7% (13.7–66.0%)	58.3% (36.9–77.1%)
Over 55	19.4% (11.2–31.5%)	12.5% (3.7–34.5%)	0%	16.7% (5.9–38.9%)
Sex				
Male	43.5% (31.5–56.4%)	33.3% (16.7–55.5%)	42.9% (18.3–71.6%)	54.2% (33.2–73.7%)
Female	56.5% (43.6–68.5%)	66.7% (44.5–83.3%)	57.1% (28.4–81.7%)	45.8% (26.3–66.8%)
Indigenous identity				
Non-Indigenous person	3.2% (0.8–12.4%)	8.3% (1.9–30.2%)	0%	0%
Indigenous person	96.8% (87.6–99.2%)	91.7% (69.8–98.1%)	100%	100%
Treatment				
Over-the-counter medications	32.3% (21.6–45.2%)	16.7% (5.9–38.9%)	50.0% (23.2–76.8%)	37.5% (19.8–59.4%)
Prescribed medications	1.6% (0.2–11.1%)	0%	7.1% (0.7–44.1%)	0%
Traditional medications	0%	0%	0%	0%
Visited clinic or hospital	4.8% (1.5–14.4%)	0%	7.1% (0.7–44.1%)	8.3% (1.9–30.2%)
Severity				
Mean number of diarrhoea on worst day	2.94 [1–6]	1.38 [1–2]	4.00 [1–16]	3.81 [1–6]
Mean number of times vomiting on worst day	2.15 [1–6]	1.00 [1–1]	3.00 [2–4]	2.38 [1–6]
Duration of illness				
Mean duration of AGI illness (days)	2.30 [1–7]	1.00 [1–1]	1.00 [1–1]	3.17 [2–7]
Mean duration of diarrhoea symptoms (days)	1.63 [1–7]	1.00 [1–1]	1.00 [1–1]	2.89 [2–7]
Mean duration of vomiting symptoms (days)	1.8 [1–7]	1.00 [1–1]	1.00 [1–1]	2.33 [1–7]
Missed activities				
Mean duration of missed usual activities (days)	0.33 [0–4]	0.04 [0–1]	0.21 [0–1]	0.67 [0–4]
Mean duration of missed work (days)	0.21 [0–4]	0.21 [0–2]	0.21 [0–1]	0.21 [0–4]
Mean duration of missed traditional activities (days)	0.16 [0–3]	0.04 [0–1]	0.00 [0–0]	0.38 [0–3]
Mean duration of missed work for caregiver (days)	0.16 [0–1]	0.04 [0–1]	0.00 [0–0]	0.00 [0–0]

**Table II:** Treatment, duration and severity of acute gastrointestinal illness in Iqaluit, Nunavut, in September 2012 and May 2013

	Iqaluit			
	
	September and May combined			
	
Treatment, duration and severity	All cases % (95% CI) Mean [range]	Mild cases % (95% CI) Mean [range]	Moderate cases % (95% CI) Mean [range]	Severe cases % (95% CI) Mean [range]
Age				
0–19	18.4% (12.5–26.3%)	16.7% (4.8–44.1%)	14.0% (6.2–28.5%)	21.9% (13.2–34.0%)
20–55	63.2% (54.3–71.3%)	55.6% (30.9–77.8%)	72.1% (56.3–83.8%)	59.4% (46.7–70.9%)
Over 55	18.4% (12.5–26.3%)	27.8% (10.9–54.7%)	14.0% (6.2–28.5%)	18.8% (10.8–30.5%)
Sex				
Male	36.0% (28.0–44.9%)	55.6% (30.9–77.8%)	32.6% (19.9–48.4%)	32.8% (22.2–45.5%)
Female	64.0% (55.1–72.0%)	44.4% (22.2–69.1%)	67.4% (51.6–80.1%)	67.2% (54.5–77.8%)
Indigenous identity				
Non-Indigenous person	32.3% (24.5–41.1%)	38.9% (18.2–64.5%)	40.5% (26.3–56.4%)	25.0% (15.7–37.4%)
Indigenous person	67.7% (58.9–75.5%)	61.1% (35.5–81.8%)	59.5% (43.6–73.7%)	75.0% (62.6–84.3%)
Treatment				
Over-the-counter medications	45.6% (37.0–54.5%)	27.8% (10.9–54.7%)	34.9% (21.8–50.7%)	57.8% (45.1–69.5%)
Prescribed medications	8.8% (4.9–15.3%)	5.6% (0.6–35.5%)	9.3% (3.4–23.1%)	9.4% (4.2–19.7%)
Traditional medications	10.4% (6.1–17.2%)	11.1% (2.4–38.9%)	9.3% (3.4–23.1%)	10.9% (5.2–21.6%)
Visited clinic or hospital	16.9% (11.2–24.7%)	5.9% (0.7–37.3%)	16.3% (7.7–31.1%)	20.3% (12.0–32.3%)
Severity				
Mean number of diarrhoea on worst day	3.80 [1–6]	1.54 [1–2]	4.17 [2–6]	4.13 [1–6]
Mean number of times vomiting on worst day	3.16 [1–6]	1.00 [1–1]	3.4 [1–6]	3.37 [1–6]
Duration of illness				
Mean duration of AGI illness (days)	3.17 [1–7]	1.00 [1–1]	1.00 [1–1]	3.83 [2–7]
Mean duration of diarrhoea symptoms (days)	2.34 [1–7]	1.00 [1–1]	1.00 [1–1]	3.35 [2–7]
Mean duration of vomiting symptoms (days)	1.91 [1–7]	1.00 [1–1]	1.00 [1–1]	2.67 [1–7]
Missed activities				
Mean duration of missed usual activities (days)	1.01 [0–7]	0.21 [0–2]	0.88 [0–4]	1.29 [0–7]
Mean duration of missed work (days)	0.83 [0–7]	0.40 [0–3]	0.69 [0–4]	1.03 [0–7]
Mean duration of missed traditional activities (days)	0.75 [0–7]	0.12 [0–2]	0.51 [0–7]	1.08 [0–7]
Mean duration of missed work for caregiver (days)	0.31 [0–7]	0.17 [0–2]	0.23 [0–3]	0.41 [0–7]

### AGI symptoms and severity

**Fig. 3:**
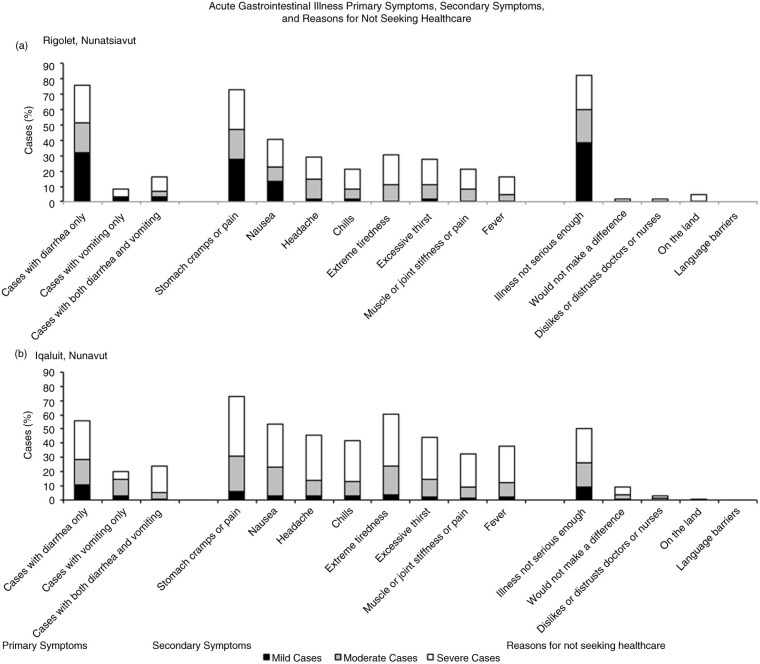
An overview of acute gastrointestinal illness case counts of primary symptoms, secondary symptoms, and reasons for not seeking healthcare by severity for Rigolet, Nunatsiavut (a), and Iqaluit, Nunavut (b), in September 2012 and May 2013.

In both communities, most cases were classified as severe (38.7% in Rigolet; 51.2% in Iqaluit), followed by moderate, and mild AGI; cases seeking treatment were most often in the severe category (Tables I and II). In Iqaluit, severe symptoms were reported by more women (67.2% of total severe cases) than men, and by more Indigenous people (75.0% of total severe cases) than non-Indigenous people (Tables I and II). The treatment, duration, productivity impacts and demographics of cases are stratified by severity of illness in Table I and II. Primary and secondary AGI symptoms are stratified by severity and presented in Fig. 3.

### AGI and OTC medication use

In Rigolet, approximately one-third of cases took OTC medications (Tables I and II). Univariable exact logistic regression analysis found increased odds of OTC medication use was unconditionally associated with secondary symptoms (headache, extreme tiredness, chills and muscle pain), as well as lower education levels of the household head (Table III). In Iqaluit, many cases reported taking OTC medications for their illness and few cases reported taking traditional medicines (Tables I and II). In Iqaluit, the final multivariable exact logistic regression model found increased odds of taking OTC medication were associated with missing paid employment due to illness and severe AGI symptoms (Table IV).

**Table III:** Univariable exact logistic regression, examining the effects of predictor variables on the odds of taking over-the-counter (OTC) medication for AGI in Rigolet, Nunatsiavut (September 2012 and May 2013)

Rigolet OTC medication use (September and May)					

				Univariable exact logistic results	
				
Predictor variable		n	Odds ratio	p*	95% CI
No. of secondary symptoms reported	None	36	ref.		
	1	9	4.946	0.043	0.830–32.521
	2+	17	3.581	0.053	0.869–15.560
Education level of household head	High school or less	41	ref.		
	Post-secondary	20	0.254	0.046	0.041–1.088
Extreme tiredness	No	41	ref.		
	Yes	19	4.740	0.009	1.302–18.583
Headache	No	41	ref.		
	Yes	18	11.910	0.001	2.898–58.550
Muscle pains	No	45	ref.		
	Yes	13	4.273	0.026	1.005–20.226
Chills	No	45	ref.		
	Yes	13	6.680	0.005	1.509–35.916

* Score method for estimating p-values does not assume a symmetrical distribution for discrete data. p<0.05 was considered significant.

**Table IV:** Univariable exact logistic regression (for variables with p<0.20) and final multivariable logistic regression results, examining the effects of predictor variables on the odds of taking over-the-counter (OTC) medication for AGI in Iqaluit, Nunavut (September 2012 and May 2013)

Iqaluit OTC medication use (September and May)								

				Univariable exact logistic results		Final multivariable exact logistic results		
					
Predictor variable		n	Odds Ratio	p*	95% CI	Odds Ratio	p*	95% CI
Variables in the final multivariable model								
Missed paid employment due to AGI	No	71	ref.			ref.		
	Yes	51	5.024	<0.001	2.190–11.986	5.649	<0.001	2.357–14.417
Severity of illness	Mild cases	18	0.285	0.777	0.071–0.980	0.216	0.041	0.057–1.022
	Moderate cases	43	0.394	0.033	0.162–0.933	0.344	0.019	0.124–0.902
	Severe Cases	64	ref.			ref.		
Variables considered in building the multivariable model								
Chill	No	72	ref.			–	–	–
	Yes	52	3.658	0.001	1.639–8.411	–	–	–
Extreme thirst	No	67	ref.			–	–	–
	Yes	55	2.004	0.069	0.917–4.441	–	–	–
Fever (self-reported)	No	77	ref.			–	–	–
	Yes	47	2.421	0.026	1.089–5.492	–	–	–
Headache	No	64	ref.			–	–	–
	Yes	57	1.842	0.103	0.843–4.079	–	–	–
Muscle pains	No	79	ref.			–	–	–
	Yes	41	1.967	0.086	0.860–4.568	–	–	–
Nausea	No	55	ref.			–	–	–
	Yes	67	2.370	0.028	1.071–5.369	–	–	–
Stomach cramps	No	31	ref.			–	–	–
	Yes	91	1.955	0.145	0.777–5.201	–	–	–
Vomited	No	70	ref.			–	–	–
	Yes	55	2.850	0.006	1.302–6.379	–	–	–
Missed daily activities due to AGI	No	58	ref.			–	–	–
	Yes	62	0.517	0.098	0.232–1.133	–	–	–
Missed traditional activities due to AGI	No	95	ref.			–	–	–
	Yes	28	2.302	0.083	0.902–6.094	–	–	–
Age	0–19	23	ref.			–	–	–
	20–55	79	0.330	0.031	0.107–0.947	–	–	–
	Over 55	23	0.589	0.550	0.150–2.216	–	–	–

* Score method for estimating p-values does not assume a symmetrical distribution for discrete data. p<0.05 was considered significant.

### Healthcare use

**Fig. 4:**
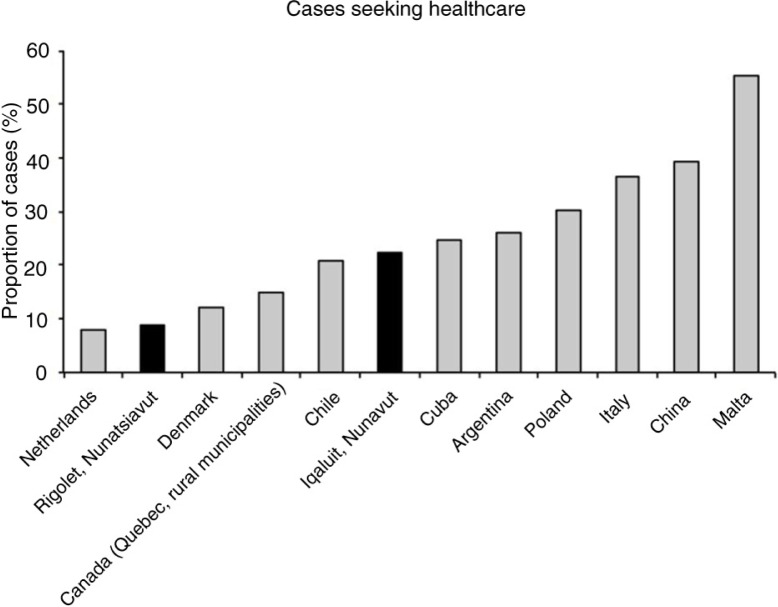
Proportion of cases seeking healthcare for acute gastrointestinal illness (case definition: 3 or more loose stools/day and/or vomiting in the past 28 days) for Netherlands (7), Rigolet, Denmark (29), Quebec (22), Chile (26), Iqaluit, Cuba (28), Argentina (21), Poland (23), Italy (6), China (24) and Malta (20). Note: To compare results to international studies, Rigolet and Iqaluit proportions are based on May survey data (28-day recall) using a stricter case definition (September data are precluded).

**Fig. 5:**
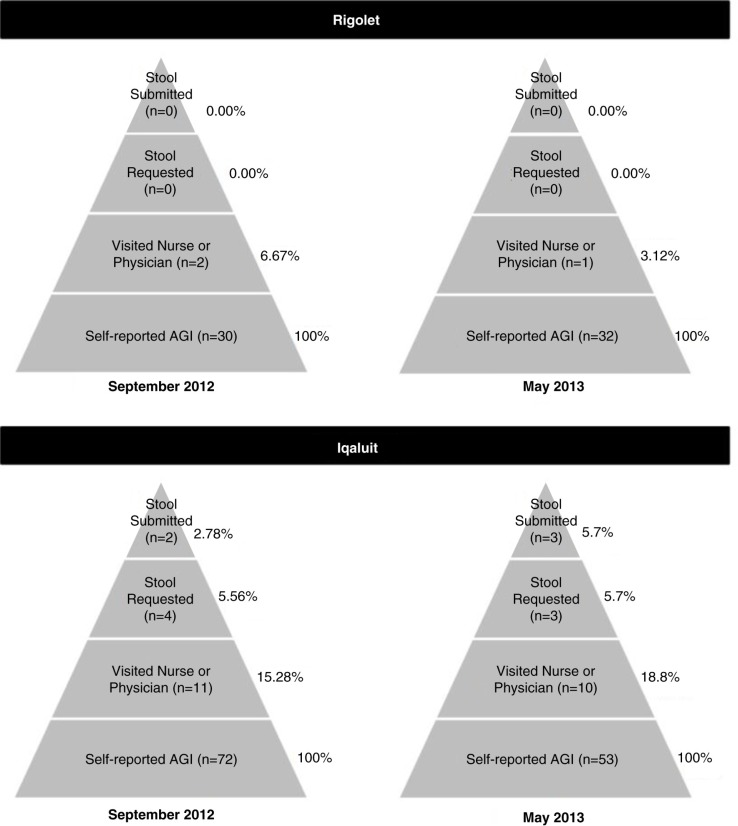
The under-reporting pyramids for acute gastrointestinal illness in Rigolet, Nunatsiavut, in September 2012 and May 2013, and for Iqaluit, Nunavut, in September 2012 and May 2013.

In Rigolet, it was rare for cases to visit the health clinic for their illness, and of the 3 cases who visited the clinic, none were asked to submit stool samples (Tables I and II; Figs. 4 and 5). Therefore, statistical analyses to identify potential predictors of healthcare use in Rigolet were precluded. In Iqaluit, some cases went to the hospital for treatment of AGI (Tables I and II; Figs. 4 and 5). Of the 21 cases who visited a clinic in Iqaluit, 7 (33.3%) were asked to submit a stool sample and 5 complied (71.4%) (Figs. 4 and 5). In Iqaluit, the odds of visiting the clinic or hospital for AGI was associated with missing traditional activities due to AGI symptoms, as well as taking OTC medication for AGI (Table V). The odds of a healthcare provider requesting a stool sample was higher for Indigenous cases than non-Indigenous cases, when controlling for diarrheal symptoms (OR=8.4; n=21; 95% CI=1.1–87.6; p=0.03).

**Table V:** Univariable exact logistic regression (for variables with p<0.20) and final multivariable logistic regression results, examining the effects of predictor variables on the odds of visiting a health clinic or hospital for AGI in Iqaluit, Nunavut (September 2012 and May 2013)

Iqaluit healthcare use (September and May)								

			Univariable logistic results			Final multivariable logistic results		
				
Predictor variable		n	Odds ratio	p*	95% CI	Odds ratio	p*	95% CI
Variables in the final multivariable model								
Missed traditional activities due to AGI	No	95	ref.					
	Yes	28	4.591	0.003	1.479–14.440	3.822	0.012	1.180–12.463
Took OTC medication for AGI	No	68	ref.					
	Yes	57	4.778	0.005	1.522–18.001	3.882	0.018	1.176–15.090
Variables considered in building the multivariable model								
Vomited	No	70	ref.			–	–	–
	Yes	55	4.055	0.0071	1.348–13.870	–	–	–
Food secure	No	57	ref.			–	–	–
	Yes	68	0.360	0.041	0.122–1.044	–	–	–
Missed paid employment due to AGI	No	71	ref.			–	–	–
	Yes	51	2.629	0.077	0.912–8.050	–	–	–
Household owns a vehicle	No	47	ref.			–	–	–
	Yes	76	0.485	0.144	0.166–1.396	–	–	–

* Score method for estimating p-values does not assume a symmetrical distribution for discrete data. p<0.05 was considered significant.

## Discussion

This cross-sectional survey facilitated the characterization of self-reported AGI symptoms and severity, and an examination of AGI factors associated with OTC medication use and healthcare use in 2 Inuit communities. Despite the higher self-reported incidence of AGI previously reported in Rigolet and Iqaluit (14) (Fig. 1), this study found lower levels of healthcare and OTC medication use for AGI symptoms compared to other areas in Canada (4) and abroad (6, 20, 21, 23, 24, 28) (Fig. 4). Healthcare use rates for AGI in Rigolet were among the lowest reported rates in the literature (6, 20, 21, 23, 24, 28); however, in Iqaluit, the proportion of cases visiting healthcare providers was lower than some studies (6, 20, 21, 23, 24, 28), but higher than others (7, 22, 26, 29). The trend of lower healthcare use for AGI symptoms was similar to that reported for other health outcomes in Indigenous communities in Canada and abroad, which found significantly lower healthcare access and use rates in Indigenous populations compared to non-Indigenous communities in the same country (2, 30). Furthermore, a study in British Columbia Canada found lower levels of healthcare use for AGI in rural regions than semi-urban and urban regions (4). There are unique factors that influence healthcare use and demand in Indigenous and rural communities, including the social and financial costs of leaving the community for specialized services, cultural differences, language barriers, racism and difficulties associated with travel (2, 13, 30, 31). Another potential barrier to AGI healthcare use in Iqaluit among Inuit, might be the fear of long wait times at the hospital. When community health centers experience staffing shortages, wait times can be long for individuals with illnesses not perceived to be “serious” or “urgent”. During such times, people are occasionally advised to wait until the next available clinic if their symptoms are minor (32). Some individuals may also fear contracting a new hospital acquired infection, or spreading their AGI infection to others during prolonged stays in crowded hospital wait rooms. In addition to experiencing linguistic barriers (absence of an interpreter) that may disable communication of their condition, Inuit have questioned the thoroughness and effectiveness of the diagnosis and treatment received from health care professionals (32). Perceptions of the quality and responsiveness of local healthcare services may influence whether Inuit in Iqaluit decide to seek medical attention for an AGI-related condition. Inuit sometimes consult Elder family members for advice on how best to treat children with stomach illnesses (32). This advice may be another factor in determining whether parents seek medical attention or decide to self manage AGI symptoms in children. These factors may have contributed to lower AGI-related healthcare use in this study, which warrants further investigation to continue improving the accessibility and suitability of healthcare provision in the north.

Unlike other studies in Canada (4) and USA (5), the severity of symptoms was not associated with healthcare use in Iqaluit; however, missing paid employment and traditional activities were associated with increased healthcare use. This finding reflects pervious research that suggested some Inuit do not characterize AGI severity by physiological symptoms (i.e. Acute Clinical Model of Health), but rather in terms of lost productivity (i.e. Role Performance Model of Health) (15, 33). The goal of seeking healthcare treatment in the Acute Clinical Model is to reduce and relieve symptoms, which is common in urban, Western settings. Whereas, the goal of seeking healthcare treatment in the Role Performance Model is to increase functional ability to fulfil work, family and community roles and responsibilities, which is more common in rural and Indigenous contexts (15, 33). These differences in how severity of AGI is defined and the goal of seeking healthcare might help explain why severity of AGI symptoms were associated with healthcare use in other studies but not in Iqaluit, as well as why lost productivity was associated with healthcare use in Iqaluit, but not in other studies. It is also important to note that in Iqaluit, many employers require a note from a healthcare provider if an individual misses more than 3 days of work. Furthermore, taking OTC medication was also associated with healthcare use, which is similar to results in Canada (4) and could suggest that cases attempted to self-treat AGI before they decided to interact with the healthcare system. Alternatively, it could suggest that the case took OTC medication after the clinic visit at the recommendation of a healthcare provider. Unlike past studies, age and sex were not associated with healthcare use (5). Taken together, our results could suggest that factors associated with healthcare use in the North are different than the South, which could result in differential biases in types of cases captured in national surveillance system data. That is, in the South, studies suggest that children, elderly and cases with severe symptoms are over-represented in clinic and hospital AGI data (4, 5); in the North, our results suggest that cases with lost productivity are more likely to use healthcare services and are thus over-represented in clinic or hospital records. This difference should be considered when using clinic records, hospital records, or surveillance data in examining and comparing the burden of AGI in the North to other locales.

The use of OTC medications within a community varies according to perceptions, beliefs and attitudes about the effectiveness and usefulness of OTC medication for treating symptoms, as well as potential side-effects (15). While this varies at the individual level, previous research at the population level has indicated that sales rates of OTC medications (e.g. non-prescribed anti-diarrheal and anti-nauseants) can reflect the occurrence of AGI at the community-level in Canada (34). We found that the rates of AGI-related OTC medication use were similar to Ontario (35) but lower than British Columbia (4). Similar to past studies (36), we found that missing paid employment due to AGI, severity of primary AGI symptoms, secondary symptoms (e.g. headache, muscle pains, chills) and younger ages were associated with increased odds of OTC medication use.

Very few cases reported using traditional medicine to treat AGI symptoms. Perhaps Inuit traditional remedies are no longer regularly used for AGI in the 2 communities in this study; however, other research identified the use of seal meat and oil (37) and medicinal plants (38) by Baffin Island Inuit to treat AGI symptoms. One study found that traditional Inuit medicine was reported to be available by 11% of respondents in Nunatsiavut, and 16% of respondents in Nunavut (39). Considering the higher availability of traditional medicine in Nunavut (39), the low use of Inuit traditional medicine to treat AGI symptoms in Iqaluit could reflect response bias or the more “urbanized” nature of Iqaluit compared to the other much smaller communities in Nunavut.

For AGI to be captured by a surveillance system, the case must come into contact with the healthcare system, the healthcare provider must request a stool sample and the case must comply with the request. Based on the low number of cases seeking healthcare for AGI symptoms and the infrequency of submitting stool samples for laboratory testing, our study suggests that AGI in Rigolet and Iqaluit is likely substantially under-ascertained in national surveillance records. But, of those who sought medical care in Iqaluit, the proportion of cases from whom stools samples were requested was 35% and 71% of cases complied, which is higher than that reported in other studies in Canada (4) and the USA (5). Unlike other studies (4, 40), we did not find any significant associations between healthcare providers requesting stools samples and secondary symptoms, age, or OTC medication use. When controlling for diarrheal symptoms, we found increased odds of healthcare providers requesting stool samples from Indigenous cases. However, there are a number of demographic and clinical presentation considerations that physicians take into consideration when deciding whether or not to request a stool sample (4, 41), and our results are very exploratory in nature due to the small sample size, and more research is warranted.

This study had several limitations. Firstly, this study followed a cross-sectional design relying on self-reported information with no pathogen testing, which could over- or under-estimate AGI healthcare seeking behaviours and OTC medication use due to recall bias. However, we selected a 14-day recall period to minimize potential recall bias based on local partner advice about reliably recalling health care seeking behaviours. Furthermore, the AGI case definition that was used is very sensitive and captures symptoms caused by various conditions, as well as infectious pathogens; as such, it is impossible to determine how much of the burden was caused by infection that could be captured by national surveillance or other acute conditions that would not be captured by national surveillance. Nonetheless, using this case definition facilitates comparison with data from Canada (4, 10) and also allows future re-analysis using more restrictive definitions for international comparisons (6, 20, 21, 23, 24–26, 28). Secondly, the p-values presented herein should be considered exploratory in nature due to the small sample size and power. Thirdly, data were captured at 2 points in time; while these results provide insights into the fall and spring season in 2012 and 2013, the results might not be representative of other seasons or times of year and do not represent seasonal trends over time. Furthermore, this study was conducted in 2 Inuit communities to explore 2 different regions, a small and large community, and an urban and rural setting; however, caution should be used in generalizing the study results given the variation and diversity among Inuit communities across the North. The response rates in Iqaluit varied between September and May surveys; however, we did not collect data on reasons why individuals declined participation. We hypothesize that this difference in response rates might be a result of better weather and travel conditions in May, which might have impacted an individual's time available and motivation to participate in the survey. Finally, while this study nearly achieved a census sample in Rigolet, the sample size was still small and thus precluded multivariable modelling for this community. Still, the high response rate and similar demographics of the survey population and the Canadian census for Rigolet is an important achievement and presents a meaningful contribution to Indigenous health literature.

Understanding what factors impact healthcare use is important to inform and improve healthcare services, and to increase the efficacy of public health surveillance. While rates of self-reported AGI were higher in Inuit communities compared to non-Inuit communities in Canada (14), there were lower rates of AGI-related healthcare use in Inuit communities compared to other regions in Canada. Furthermore, the factors associated with healthcare use were different in Iqaluit than other studies in Canada and USA (4, 5). As such, the rates and predictors of healthcare use for a given disease can differ between Inuit and non-Inuit communities, and caution should be exercised in making comparisons in health outcomes between Inuit and non-Inuit communities based solely on clinic records and healthcare use.

## References

[1] King M, Smith A, Gracey M (2009). Indigenous health part 2: the underlying causes of the health gap. Lancet.

[2] Marrone S (2007). Understanding barriers to health care: a review of disparities in health care. Int J Circumpolar Health.

[3] Tjepkema M (2002). The Health of the off-reserve aboriginal population. Stat Can.

[4] MacDougall L, Majowicz S, Doré K, Flint J, Thomas K, Kovacs S (2008). Under-reporting of infectious gastrointestinal illness in British Columbia, Canada: who is counted in provincial communicable disease statistics?. Epidemiol Infect.

[5] Scallan E, Jones TF, Cronquist A, Thomas S, Frenzen P, Hoefer D (2006). Factors associated with seeking medical care and submitting a stool sample in estimating the burden of foodborne illness. Foodborne Pathog Dis.

[6] Scavia G, Baldinelli F, Busani L, Caprioli A (2012). The burden of self-reported acute gastrointestinal illness in Italy: a retrospective survey, 2008–2009. Epidemiol Infect.

[7] Doorduyn Y, Van Pelt W, Havelaar H, Havelaar AH (2012). The burden of infectious intestinal disease (IID) in the community: a survey of self-reported IID in The Netherlands. Epidemiol Infect.

[8] Thomas K, Murray R, Flockhart L, Pintar K, Pollari F, Fazil A (2013). Estimates of the burden of foodborne illness in Canada for 30 specified pathogens and unspecified agents, Circa 2006. Foodborne Pathog Dis.

[9] Roy SL, Beach MJ, Scallan E (2006). The rate of acute gastrointestinal illness in developed countries. J Water Health.

[10] Majowicz S, Edge VL, Fazil A, McNab WB, Doré KS, Sockett PN (2005). Estimating the under-reporting rate for infectious gastrointestinal illness in Ontario. Can J Public Health.

[11] Harper SL, Edge VL, Schuster-Wallace CJ, Ar-Rushdi M, McEwen SA (2011). Improving aboriginal health data capture: evidence from a health registry evaluation. Epidemiol Infect.

[12] Pardhan-Ali A, Berke O, Wilson J, Edge VL, Furgal C, Reid-Smith R (2012). A spatial and temporal analysis of notifiable gastrointestinal illness in the Northwest Territories, Canada, 1991–2008. Int J Health Geogr.

[13] Pardhan-Ali A, Wilson J, Edge VL, Furgal C, Reid-Smith R, Santos M (2012). A descriptive analysis of notifiable gastrointestinal illness in the Northwest Territories, Canada, 1991–2008. BMJ Open.

[14] Harper SL, Edge VL, Ford JD, Thomas MK, Pearl DL, Shirley J (2015). Acute gastrointestinal illness in two Inuit communities: burden of illness in Rigolet and Iqaluit, Canada. Epidemiol Infect.

[15] Harper SL, Edge VL, Ford J, Thomas MK, Rigolet Inuit Community Government, IHACC Research Group (2015). Lived experience of acute gastrointestinal illness in Rigolet, Nunatsiavut: just suffer through it. Soc Sci Med.

[16] Statistics Canada (2007). 2006 Community Profiles. http://www12.statcan.ca/english/census06/data/profiles/community/Index.cfm?Lang=E.

[17] Henson SJ, Majowicz SE, Masakure O, Sockett PN, MacDougall L, Edge VL (2008). Estimation of the costs of acute gastrointestinal illness in British Columbia, Canada. Int J Food Microbiol.

[18] Bickel G, Nord M, Price C, Hamilton W, Cook J (2000). Guide to measuring household food security, revised 2000.

[19] Dohoo I, Martin S, Stryhn H (2012). Methods in epidemiologic research.

[20] Gauci C, Gilles H, O'brien S, Mamo J, Stabile I, Ruggeri FM (2007). The magnitude and distribution of infectious intestinal disease in Malta: a population-based study. Epidemiol Infect.

[21] Thomas MK, Perez E, Majowicz SE, Reid-Smith R, Albil S, Monteverde M (2010). Burden of acute gastrointestinal illness in Gálvez, Argentina, 2007. J Health Popul Nutr.

[22] Febriani Y, Levallois P, Gingras S, Gosselin P, Majowicz SE, Fleury MD (2010). The association between farming activities, precipitation, and the risk of acute gastrointestinal illness in rural municipalities of Quebec, Canada: a cross-sectional study. BMC Public Health.

[23] Baumann-Popczyk A, Sadkowska-Todys M, Rogalska J, Stefanoff P (2012). Incidence of self-reported acute gastrointestinal infections in the community in Poland: a population-based study. Epidemiol Infect.

[24] Ho SC, Chau PH, Fung PK, Sham A, Nelson E, Sung J (2010). Acute gastroenteritis in Hong Kong: a population-based telephone survey. Epidemiol Infect.

[25] Hall GV, Kirk MD, Ashbolt R, Stafford R, Lalor K (2006). Frequency of infectious gastrointestinal illness in Australia, 2002: regional, seasonal and demographic variation. Epidemiol Infect.

[26] Thomas MK, Perez E, Majowicz SE, Reid-Smith R, Olea A, Diaz J (2011). Burden of acute gastrointestinal illness in the Metropolitan region, Chile, 2008. Epidemiol Infect.

[27] Messier V, Lévesque B, Proulx J-F, Ward BJ, Libman M, Couillard M (2007). Zoonotic diseases, drinking water and gastroenteritis in Nunavik: a brief portrait.

[28] Prieto PA, Finley RL, Muchaal PK, Guerin MT, Isaacs S, Domínguez AC (2009). Burden of self-reported acute gastrointestinal illness in Cuba study design. J Health Popul Nutr.

[29] Müller L, Korsgaard H, Ethelberg S (2012). Burden of acute gastrointestinal illness in Denmark 2009: a population-based telephone survey. Epidemiol Infect.

[30] Newbold KB (1997). Aboriginal physician use in Canada: location, orientation, and identity. Health Econ.

[31] Islam R, Sheikh MA (2010). Cultural and socio-economic factors in health, health services and prevention for indigenous people. Antrocom Online JAnthropol.

[32] Edgecombe N (2006). Healthcare Decision making in Kugaaruk, Nunavut. Doctoral Thesis.

[33] Weinert C, Long KA (2013). Understanding the health care needs of rural families. Fam Relat.

[34] Edge VL, Pollari F, Ng LK, Michel P, McEwen SA, Wilson JB (2006). Syndromic surveillance of norovirus using over-the-counter sales of medications related to gastrointestinal illness. Can J Infect Dis Med Microbiol.

[35] Sargeant JM, Majowicz SE, Snelgrove J (2008). The burden of acute gastrointestinal illness in Ontario, Canada, 2005–2006. Epidemiol Infect.

[36] Frosst GO, Majowicz SE, Edge VL (2006). Factors associated with the use of over-the-counter medications in cases of acute gastroenteritis in Hamilton, Ontario. Can J Public Health.

[37] Borré K (2012). The healing power of the seal: the meaning of Inuit health practice and belief. Arctic Anthropol.

[38] Black PL, Arnason JT, Cuerrier A (2008). Medicinal plants used by the Inuit of Qikiqtaaluk (Baffin Island, Nunavut). Botany.

[39] ITK (2006). Naasautit: Inuit health statistics. http://www.inuitknowledge.ca/naasautit.

[40] Imhoff B, Morse D, Shiferaw B, Hawkins M, Vugia D, Lance-Parker S (2004). Burden of self-reported acute diarrheal illness in FoodNet surveillance areas, 1998–1999. Clin Infect Dis.

[41] Edge VL, Odoi A, Fyfe M, MacDougall L, Majowicz SE, Doré K (2007). Physician diagnostic and reporting practices for gastrointestinal illnesses in three health regions of British Columbia. Can J Public Health.

